# Multi-Omics Mendelian Randomization and Clinical Validation Implicate NLRP6 as a Candidate Autophagy-Related Gene in Systemic Lupus Erythematosus

**DOI:** 10.3390/genes17040466

**Published:** 2026-04-16

**Authors:** Daan Nie, Jianguo Yin, Wei Tu, Kecheng Huang, Jing Wan, Yikai Yu, Bei Wang, Yu Chen, Shengyan Lin, Zhipeng Zeng

**Affiliations:** 1 Department of Cardiology, Hunan University of Medicine General Hospital, Huaihua 418000, China; nda2008@163.com (D.N.); yinjianguo1121@163.com (J.Y.); 2 Department of Rheumatology and Immunology, Tongji Hospital, Tongji Medical College, Huazhong University of Science and Technology, Wuhan 430030, China; tuwei@tjh.tjmu.edu.cn (W.T.); peng5760242@126.com (J.W.); yuyikai@tjh.tjmu.edu.cn (Y.Y.); wangb_rd@tjh.tjmu.edu.cn (B.W.); yuchen0910@tjh.tjmu.edu.cn (Y.C.);; 3 Department of Obstetrics and Gynecology, Tongji Hospital, Tongji Medical College, Huazhong University of Science and Technology, Wuhan 430030, China; huangkc@hust.edu.cn

**Keywords:** systemic lupus erythematosus, autophagy, multi-Omics, mendelian randomization analysis, quantitative trait loci, *NLRP6*

## Abstract

**Background/Objectives:** Autophagy plays a role in systemic lupus erythematosus (SLE) pathogenesis. Nevertheless, the specific genetic determinants underpinning this process remain poorly characterized. Summary data-based Mendelian randomization (SMR) analysis was therefore utilized to pinpoint autophagy-related genes associated with SLE risk. **Methods:** We analyzed 700 autophagy-related genes, integrating methylation quantitative trait loci (mQTL), expression QTL (eQTL) from blood and relevant tissue, and protein QTL (pQTL) data with genome-wide association studies (GWAS) data on SLE from the IEU dataset (discovery). GWAS data from FinnGen and the GWAS Catalog were used as replication datasets. Colocalization analysis identified shared genetic variants. Blood samples from 10 healthy control and 20 SLE patients were collected and analyzed for the expression of candidate genes. **Results:** Our SMR analysis identified suggestive associations between *NLRP6* expression (OR = 0.528, 95%CI = 0.291–0.96) and p27Kip1 protein abundance (OR = 0.269, 95%CI = 0.08–0.904) with SLE susceptibility in the discovery cohort, supported by colocalization evidence. Additionally, we found that the methylation of the *NLRP6* promoter (cg06432119) was significantly increased, while *NLRP6* expression and p27Kip1 level were significantly decreased in SLE patients compared to controls. Furthermore, *NLRP6* mRNA expression was significantly negatively correlated with the SLE severity (SLEDAI-2000). **Conclusions:** These findings not only prioritized candidate genes via SMR analysis but also provided evidence of epigenetic dysregulation of *NLRP6* and its correlation with disease activity in SLE, thereby offering novel insights into the underlying mechanisms.

## 1. Introduction

Systemic lupus erythematosus (SLE) is a chronic autoimmune disorder characterized by a dysregulated immune system that attacks the body’s own tissues and organs. This results in systemic inflammation and tissue damage [1], with common clinical manifestations including fatigue, joint pain, skin rashes, and kidney dysfunction [2]. It disproportionately affects non-white women, with onset typically occurring between the ages of 20 and 40 [3]. Globally, SLE occurs at a rate of 1.5 to 11 cases per 100,000 person/year, while existing prevalence estimates span a remarkably broad spectrum from 13 to 7713.5 per 100,000 individuals [4]. Patients with SLE face a two- to three-fold increase in mortality compared to the general population, primarily due to infections, renal disease, and cardiovascular disease [5]. Current treatments focus on managing symptoms and controlling inflammation with immunosuppressive drugs [6], but these therapies often cause significant side effects, and no cure exists for SLE [7]. Genetic predisposition, environmental triggers, and hormonal influences have all been implicated in this condition. However, the precise etiology continues to elude definitive characterization. Identifying genes associated with SLE could contribute to the development of more precise treatments that target the underlying mechanisms of the disease, potentially improving patient outcomes.

Autophagy plays a significant role in maintaining immune homeostasis [8]. Genetic variants in autophagy-related pathways have been linked to increased susceptibility to SLE [9]. Beyond genetics, autophagy also contributes to the clearance of dead cells, immune complexes, and intracellular debris, processes that are essential for preventing the accumulation of autoantigens [10]. In SLE, impaired autophagy compromises the body’s clearance mechanisms, which subsequently promotes immune activation and inflammation. This dysregulation is observable across key immune cells. For instance, autophagy is known to regulate monocyte differentiation and macrophage survival, and its dysfunction can exacerbate disease by driving the production of pro-inflammatory cytokines [11]. Within B cells, autophagy is upregulated during their differentiation into plasma cells, thus contributing to autoantibody overproduction [12]. Similarly, in T cells, increased autophagic flux supports their heightened activation and survival, perpetuating the autoimmune response in SLE [13]. These findings underscore the centrality of autophagy, making the identification of its regulatory genes a critical step to uncover SLE mechanisms and pave the way for targeted treatments.

Summary-data-based Mendelian Randomization (SMR) constitutes a robust analytical framework that synthesizes genome-wide association study (GWAS) findings with diverse molecular quantitative trait loci (QTL) datasets, encompassing methylation QTLs (mQTLs), expression QTLs (eQTLs), and protein QTLs (pQTLs) [14]. By leveraging genetic variants as instrumental variables, this approach facilitates the identification of candidate genes and their regulatory architectures while mitigating confounding biases. This analytical approach has demonstrated substantial utility in elucidating the molecular pathophysiology of autoimmune disorders and holds considerable promise for identifying novel therapeutic targets amenable to clinical intervention [15].

This study employed a comprehensive SMR analysis framework to identify autophagy-related genes in SLE. We systematically integrated blood-derived mQTL, eQTL, and pQTL data, along with tissue-specific eQTL data from kidney cortex and skin, with large-scale GWAS summary statistics, to elucidate the molecular mechanisms linking genetic variations to SLE pathogenesis. Initial findings were validated using independent SLE cohorts. Furthermore, we conducted integrative multi-omics analyses to decipher regulatory relationships. Collectively, our findings uncover candidate autophagy-related genes in SLE, thereby illuminating previously unrecognized pathogenic mechanisms and highlighting attractive targets for therapeutic development.

## 2. Materials and Methods

### 2.1. Ethics

This study was performed in accordance with the Declaration of Helsinki, with the protocol formally approved by the Ethics Committee of Tongji Hospital, Tongji Medical College, Huazhong University of Science and Technology (approval number: TJ-IRB202508102). Written informed consent was obtained from each volunteer prior to enrollment. The SMR analyses were performed using publicly available summary-level data from GWAS and QTL consortia. As these data were anonymized and publicly available, no additional institutional review board approval or informed consent was required for the SMR analysis.

### 2.2. Study Design

**Figure 1:**
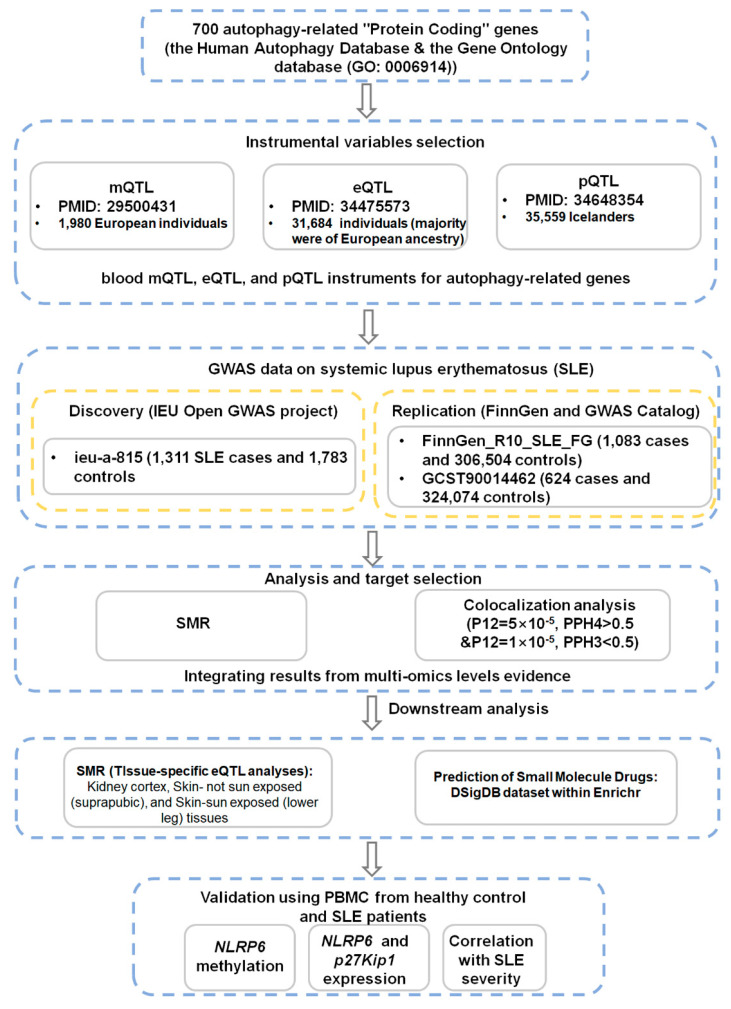
Study workflow for identifying autophagy-related genes associated with systemic lupus erythematosus (SLE).

This investigation employed a multi-phase workflow (Figure 1). Initially, SMR analysis was conducted by integrating GWAS data from the IEU OpenGWAS project (discovery cohort) and blood-based m/e/pQTL data from published sources to pinpoint potential autophagy-related genes associated with SLE. Subsequently, colocalization analyses were performed to ascertain whether shared genetic variants concurrently influenced both the molecular quantitative trait loci and disease susceptibility. Results from the discovery phase were then subjected to validation using independent SLE GWAS datasets from the FinnGen project and the GWAS Catalog. To further explore tissue-specific effects, SMR analyses were also conducted using eQTL data from kidney cortex, sun-exposed skin, and non-sun-exposed skin tissues. Subsequently, multi-omics integrative analyses spanning mQTL and eQTL were conducted to elucidate potential regulatory mechanisms. In addition, an in silico drug repurposing analysis using Enrichr and the DSigDB database was conducted to identify potential therapeutic compounds targeting SMR-prioritized genes. Finally, the methylation and expression of candidate genes were validated in peripheral blood mononuclear cells (PBMCs).

### 2.3. Data Sources of QTLs

A total of 232 genes associated with autophagy were downloaded from the Human Autophagy Database (HADb), and 579 autophagy-related genes were extracted from the Gene Ontology (GO) database (GO: 0006914) using the R package org.Hs.eg.db. After removing overlapping genes from both databases, a final autophagy-related gene set comprising 700 genes was constructed (Table S1). Blood-derived methylation quantitative trait loci were extracted from a meta-analysis of two European cohorts [16]: the Brisbane Systems Genetics Study (n = 614) and the Lothian Birth Cohorts (n = 1366). eQTL data were retrieved from the eQTLGen consortium [17], encompassing genetic data on blood gene expression from 31,684 European individuals. Blood pQTL data were sourced from a previous study [18], comprising 35,559 Icelanders.

To further investigate the potential relationships between the expression of autophagy-related genes in specific tissues and SLE susceptibility, we utilized tissue-specific eQTL summary statistics from the Genotype-Tissue Expression (GTEx) project (version 8). Considering that the kidney and skin are commonly affected organs in SLE, we specifically selected eQTL data from Kidney Cortex, Skin—Not Sun-Exposed (Suprapubic), and Skin—Sun-Exposed (Lower leg) tissues. The GTEx project offers an extensive publicly accessible repository characterizing tissue-specific gene expression patterns and regulatory mechanisms, with datasets available through the GTEx Portal.

### 2.4. SLE Outcome Datasets

The SLE discovery dataset was obtained from the IEU Open GWAS project, comprising 1311 SLE cases and 1783 controls of European ancestry. For validation, SLE datasets were sourced from the FinnGen project (R10 release), comprising 1083 cases and 306,504 controls, and the GWAS Catalog, with 624 cases and 324,074 controls, all of European ancestry (Table S2).

### 2.5. SMR Analysis

SMR coupled with heterogeneity in dependent instruments (HEIDI) tests was performed using the SMR software tool (SMR v1.3.1) to identify autophagy-related genes associated with SLE, while HEIDI was employed to distinguish pleiotropy from linkage disequilibrium (LD) by evaluating associations across multiple SNPs within genomic regions. Top associated *cis*-QTLs within a ±1000 kb window flanking each gene (*p*-value < 5.0 × 10^−8^) were analyzed [18]. SNPs with allele frequency differences exceeding 0.2 across datasets (including LD reference samples, QTL summary data, and outcome summary data) were excluded. For mQTL, eQTL, and pQTL, the permissible threshold for the proportion of such discordant variants was capped at 0.05 [16]. When discovery data lacked effect allele frequency, the R package SumStatsRehab was used to impute the missing values with the 1000 Genomes reference, and a frequency difference threshold of 0.3 was applied in the SMR analysis.

The SMR_multi method was also used, which integrates multiple genetic variants within a ±500 kb window around the probe for SNPs with a *p*-value < 5 × 10^−8^ and LD *r*^2^ < 0.9 with the top associated SNP. To control for false positives, *p* values were adjusted using the false discovery rate (FDR) method. Results with *p*_SMR < 0.05, *p*_SMR_multi < 0.05, and *p*_HEIDI > 0.01 were used for colocalization analysis and further validation.

### 2.6. Colocalization Analysis

Bayesian colocalization analysis was conducted to ascertain whether shared genetic variants underlie the observed associations [19]. Significant associations were analyzed using the R package ‘coloc’ [20] to evaluate evidence for coincident causal variants. SNPs spanning ±1000 kb of the lead cis-QTL were interrogated under 4 competing hypotheses (H0–H4): H0: no association with either trait; H1: association with only the first trait; H2: association with only the second trait; H3: association with both traits, but with different causal variants; H4: association with both traits, sharing the same causal variant. To allow weaker QTL signals to colocalize with the GWAS signal, colocalization was considered successful when the prior probability (P12), which reflects the likelihood that a shared causal variant exists, was set to 5 × 10^−5^ and the posterior probability for shared causality (PPH4) exceeded 0.5. Additionally, at P12 = 1 × 10^−5^, colocalization was successful if PPH3 was less than 0.5, ensuring that the association is not caused by different variants [21].

### 2.7. Integration of Multi-Omics Evidence

To pinpoint potential autophagy-related genes implicated in SLE, an integrative multi-omics analysis was performed spanning mQTL, eQTL, and pQTL data. Pleiotropic associations were systematically evaluated using mQTL as exposure and eQTL as outcome, or eQTL as exposure and pQTL as outcome, Prioritized associations were identified based on *p*_SMR < 0.05, *p*_SMR_multi < 0.05, and *p*_HEIDI > 0.01.

### 2.8. Prediction of Small Molecule Drugs

To identify potential therapeutic agents targeting the autophagy-related genes, we performed an in silico drug prediction analysis. This was conducted using the Enrichr web-based platform (https://maayanlab.cloud/Enrichr/, accessed on 12 April 2026), a tool for comprehensive gene set enrichment analysis [22,23,24]. Specifically, we leveraged the Drug Signatures Database (DSigDB) integrated within Enrichr. DSigDB is a comprehensive resource that curates a vast collection of gene sets representing genes transcriptionally altered by various drugs and small molecules, as previously described [25]. The list of SMR-prioritized candidate genes was uploaded to Enrichr. The platform then queried DSigDB to identify drugs or compounds whose transcriptional signatures significantly overlapped with our input gene list, thereby suggesting potential candidates for drug repurposing or novel therapeutic development in the context of SLE.

### 2.9. Clinical Sample Collection and Patient Cohort

This study enrolled a cohort comprising 20 patients diagnosed with SLE and 10 healthy control individuals. Specifically, the healthy controls were selected from individuals undergoing routine health examinations at Tongji hospital. SLE patients were included based on their fulfillment of the ACR/EULAR 2019 revised classification criteria for SLE. Notably, lupus nephritis was defined as 24 h urinary protein excretion exceeding 0.5 g according to these criteria. Individuals presenting with active infections, hematological malignancies, neoplastic diseases, or coexisting autoimmune conditions were excluded from the study cohort.

### 2.10. PBMCs Isolation

PBMCs were isolated from peripheral venous blood using density gradient centrifugation. Briefly, 4 mL of blood was centrifuged to collect the plasma, and the remaining blood cells were diluted with an equal volume of sterile PBS. The diluted blood was carefully layered over an equal volume of Ficoll-Paque in a 10 mL centrifuge tube. The tube was then centrifuged at 2500 rpm for 20 min at room temperature with rapid acceleration and slow deceleration. After centrifugation, the PBMC layer at the interface was aspirated using a pipette and transferred to a microcentrifuge tube. The cells were subsequently pelleted by centrifugation, after which the supernatant was removed and discarded.

### 2.11. DNA Methylation Analysis by Bisulfite Sequencing PCR (BSP)

Genomic DNA was extracted from the PBMC using a standard phenol–chloroform protocol, and its quality and concentration were verified by spectrophotometry, followed by bisulfite conversion of DNA. PCR amplification was conducted using bisulfite-specific primers designed to avoid CpG sites in the target regions (Table S3). The PCR products were purified by gel extraction. The purified products were ligated into a T-vector (pMD19-T, Takara, Haymarket, Australia) and transformed into T1 competent cells. After recovery and plating, positive clones were selected for plasmid extraction and Sanger sequencing. Methylation status was analyzed by comparing the bisulfite-converted sequences with the original genomic sequence using quantitative methylation analysis software.

### 2.12. RNA Extraction and Real-Time Quantitative PCR (RT-qPCR)

Total RNA was extracted from the PBMC using TRIzol reagent (Invitrogen, Carlsbad, CA, USA) according to the manufacturer’s protocol. Briefly, 1 mL of TRIzol was added per 200 μL of PBMC, followed by chloroform separation, isopropanol precipitation, and 75% ethanol washing. RNA pellets were air-dried and subsequently resuspended in RNase-free water. RNA integrity and concentration were assessed using a NanoDrop spectrophotometer. For cDNA synthesis, total RNA was reverse-transcribed using the HiScript II Q RT SuperMix (Vazyme, Nanjing, China). Quantitative PCR was performed using ChamQ Universal SYBR qPCR Master Mix (Vazyme) on an ABI7500 Real-Time PCR System, with primers listed in Table S4. All reactions were run in triplicate, and relative gene expression was calculated using the 2^−ΔΔCt^ method with GAPDH as the internal control.

### 2.13. Statistical Analysis

All statistical analyses were conducted using R software (version 4.3.0). Manhattan plots and forest plots were generated utilizing the ggplot2 and forestplot packages, respectively. The SMRLocusPlot and SMREffectPlot functions were adapted from a previously published study [26]. For clinical validation, data are presented as mean ± SEM (or median with interquartile range). Differences between two groups were analyzed using Student’s *t*-test. Correlations between variables were evaluated using Spearman’s rank correlation coefficient.

## 3. Results

### 3.1. Autophagy-Related Gene Methylation and SLE

**Figure 2:**
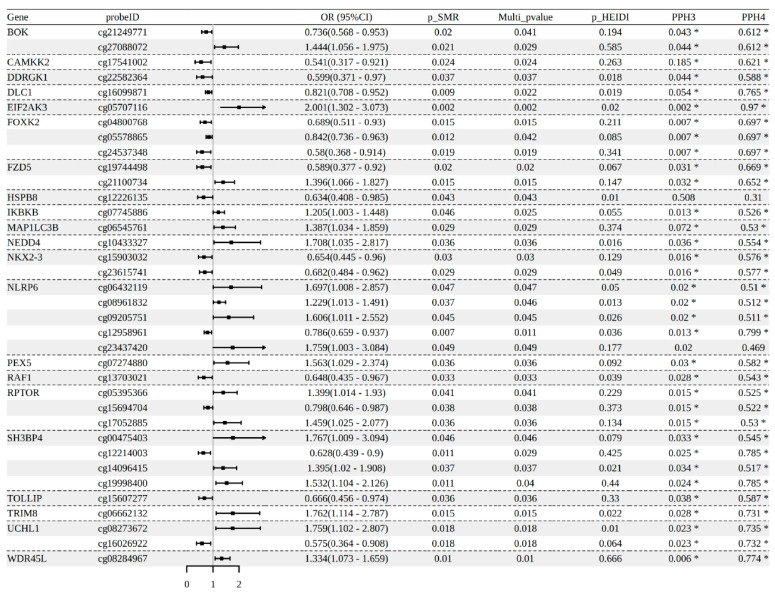
SMR analysis of autophagy-related gene methylation associated with SLE risk in the ieu-a-815 SLE dataset. The forest plot shows 36 methylation loci across 21 autophagy-related genes associated with SLE, with colocalization evidence. OR: odds ratio; 95% CI: 95% confidence intervals; PPH3, PPH4: posterior probability of colocalization. * Significant colocalization (PPH3 < 0.5 and PPH4 > 0.5).

SMR analysis identified 36 methylation sites associated with SLE across 21 autophagy-related genes in the IEU SLE cohort (Table S5; Figure 2). A total of 34 loci (near 20 genes) demonstrated colocalization evidence (PPH3 < 0.5 and PPH4 > 0.5). Notably, four methylation sites of *NLRP6* gene (cg06432119, cg08961832, cg09205751, and cg12958961) showed evidence of colocalization, supporting their potential role in SLE risk. Despite these findings, none of the *NLRP6* methylation sites were validated in external datasets (Tables S6 and S7). In contrast, both *HSPB8* and *WDR45L*, although lacking colocalization evidence, were validated in external datasets. The methylation level of cg12226135 (*HSPB8*) was negatively associated with SLE risk (OR = 0.634, 95% CI = 0.408–0.985, *p* SMR= 0.043, FDR = 0.376), which was further confirmed in the FinnGen SLE dataset. Similarly, *WDR45L* (cg08284967) showed a nominal positive correlation with SLE risk (OR = 1.334, 95% CI = 1.073–1.659, *p* SMR = 0.01, FDR = 0.224), which was validated in the GWAS Catalog SLE dataset (Table S8).

### 3.2. Autophagy-Related Gene Expression and SLE

**Figure 3:**
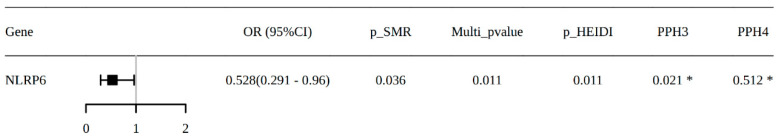
SMR analysis of autophagy-related gene expression associated with SLE risk in the ieu-a-815 SLE dataset. The forest plot shows the results of eQTL SMR analysis prioritizing *NLRP6* as associated with SLE risk. * Significant colocalization (PPH3 < 0.5 and PPH4 > 0.5).

In the IEU SLE dataset, SMR analysis identified *NLRP6* as being negatively associated with SLE risk, with colocalization evidence (OR = 0.528, 95%CI = 0.291–0.96, *p* SMR = 0.036, FDR = 0.361; Table S9; Figure 3). However, this finding was not validated in the external cohorts (Tables S10 and S11).

### 3.3. Autophagy-Related Protein Abundance and SLE

**Figure 4:**
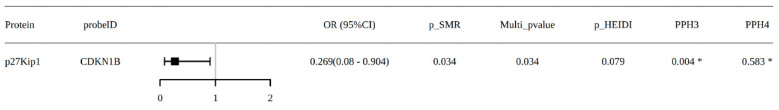
SMR analysis of autophagy-related protein abundance associated with SLE risk in the ieu-a-815 SLE dataset. The forest plot shows the results of pQTL SMR analysis prioritizing p27Kip1 protein as associated with SLE risk. * Significant colocalization (PPH3 < 0.5 and PPH4 > 0.5).

In the IEU SLE dataset, SMR analysis identified p27Kip1 as being negatively associated and colocalized with SLE susceptibility (OR = 0.269, 95%CI = 0.08–0.904, *p* SMR = 0.034, FDR = 0.346; Table S12; Figure 4). However, these findings could not be reproduced in external validation cohorts (Tables S13 and S14).

### 3.4. Integration of Multi-Omics Analyses

Integration of mQTL and eQTL summary data identified suggestive negative associations between three *NLRP6* methylation sites and gene expression (cg06432119: beta = −0.779, se = 0.131; cg09205751: beta = −0.735, se = 0.099; cg23437420: beta = −0.739, 95%CI = 0.107; Table S15 and Table 1). Considering the mQTL data, which showed that *NLRP6* (cg06432119, cg09205751, cg23437420) were positively associated with SLE risk, and the eQTL data, which indicated that *NLRP6* expression was negatively associated with SLE risk, we speculate that elevated methylation levels at these CpG sites may transcriptionally repress NLRP6, consequently augmenting susceptibility to SLE. However, integration of eQTL and pQTL summary data did not generate significant findings due to the absence of NLRP6 in the pQTL data. The Manhattan and MR locus plots demonstrated the associations between *NLRP6*-related mQTLs, eQTLs, and SLE risk (Figure S1), and the effect plots further showed the relationships between methylation, expression, and SLE risk (Figure S2), supporting the integrative analysis findings. Post hoc power analysis identified limited statistical power for detecting the observed effect sizes (38.94% for NLRP6, and 37.38% for CDKN1B), primarily reflecting the modest variance explained by the genetic instruments (R^2^ = 0.98% and 0.23%, respectively) (Table S16). The relatively low statistical power in the replication cohorts suggests that the lack of formal replication may be due to the modest sample sizes of the outcome datasets rather than a complete absence of biological effect, especially as the direction of effect remained consistent.

### 3.5. Tissue-Specific eQTL Analysis

To further elucidate the contribution of autophagy-related gene expression in specific tissues relevant to SLE, we performed SMR analyses using eQTL data from Kidney Cortex, Skin—Not Sun-Exposed (Suprapubic), and Skin—Sun-Exposed (Lower leg) tissues obtained from the GTEx v8 database, integrated with the IEU SLE discovery GWAS dataset (ieu-a-815). Prioritized associations were defined by *p*_SMR < 0.05, *p*_SMR_multi < 0.05, and *p*_HEIDI > 0.01. Unfortunately, no autophagy-related genes showed a significant association with SLE risk in the Kidney Cortex tissue eQTL analysis (Table S17). However, in the Skin—Not Sun-Exposed (Suprapubic) tissue, we identified three genes (*TOLLIP*, *HSP90AB1*, and *BAG3*) whose expression levels were significantly associated with SLE risk (Table S18). Furthermore, in the Skin—Sun-Exposed (Lower leg) tissue, five genes (*VAMP3*, *HSP90AB1*, *BAG1*, *BAG3*, and *STING1*) exhibited significant associations (Table S19). However, these tissue-specific findings did not overlap with the autophagy-related genes identified in our primary blood eQTL or pQTL analyses.

### 3.6. Prediction of Small-Molecule Drugs

We next performed an exploratory analysis to identify potential therapeutic compounds associated with autophagy-related genes implicated in SLE from our SMR analyses (*NLRP6*, *p27Kip1*, *WDR45L*, and *HSPB8*). Using the DSigDB dataset within Enrichr, we screened for drugs or compounds whose transcriptional signatures significantly overlapped with these candidate genes. This analysis identified 8 drug compounds significantly associated with *HSPB8* (adjusted *p*-value < 0.05) (Table S20).

### 3.7. Clinical Validation of SMR Candidate Genes in PBMC from SLE Patients

**Figure 5:**
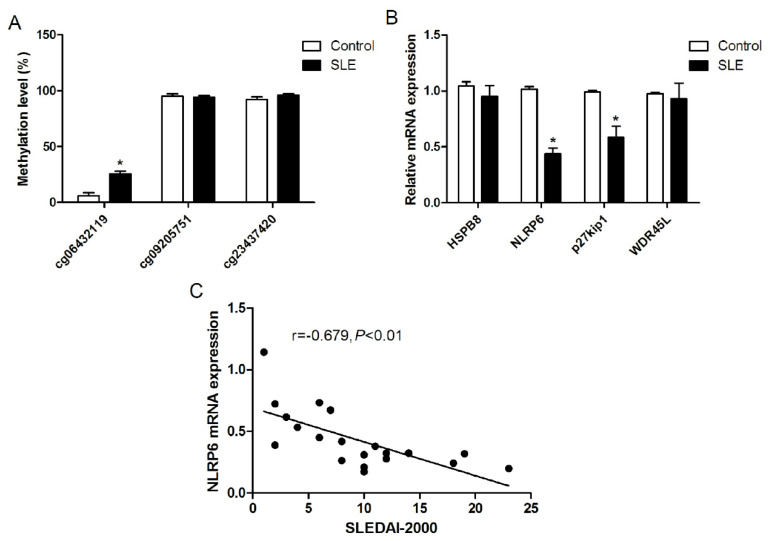
Clinical validation of candidate genes in PBMCs from SLE patients (n = 20) and healthy controls (HC, n = 10). (**A**) DNA methylation levels of three CpG sites of *NLRP6* (cg06432119, cg09205751 and cg23437420) were measured by Bisulfite Sequencing PCR (BSP). (**B**) Relative mRNA expression levels of *HSPB8*, *NLRP6*, *p27Kip1*, and *WDR45L* were measured by RT-qPCR. (**C**) Spearman correlation analysis between *NLRP6* mRNA expression and SLEDAI-2000 scores in 20 SLE patients. Data are presented as mean ± SEM. * *p* < 0.05.

The clinical characteristics of the 10 healthy controls and 20 SLE patients are summarized in Table S21. DNA methylation sequencing identified a statistically significant increase in methylation levels at the cg06432119 locus (near *NLRP6*) in SLE patients (Figure 5A). In contrast, no significant differences in methylation patterns were observed at the cg09205751 and cg23437420 loci (near *NLRP6*) between the two groups (Figure 5A). RT-qPCR analysis demonstrated significantly reduced mRNA expression levels of both *NLRP6* and *p27kip1* in SLE patients relative to controls (Figure 5B), while no significant alterations in expression were detected for *HSPB8* or *WDR45L* between these two groups. Further correlation analysis identified a significant negative correlation between *NLRP6* mRNA expression levels and disease activity as quantified by SLEDAI-2000 scores (r = −0.679, *p* < 0.01) across the 20 SLE patients (Figure 5C), suggesting that *NLRP6* expression was inversely associated with disease severity in SLE.

## 4. Discussion

In this investigation, a multi-omics SMR framework was employed to interrogate autophagy-related genes implicated in SLE. Our initial blood-based analysis identified 36 methylation sites across 21 genes. Of these, *NLRP6* expression and p27Kip1 protein abundance demonstrated associations with SLE risk in the discovery cohort. Integrative multi-omics analysis further suggested that hypermethylation at three specific *NLRP6* sites might downregulate its expression, thereby increasing SLE risk. Subsequently, preliminary clinical validation confirmed elevated methylation at the cg06432119 site and corresponding decreased expression of both *NLRP6* and *p27Kip1* in SLE patients. Notably, *NLRP6* mRNA levels exhibited a significant negative correlation with disease activity, underscoring its critical role in SLE pathogenesis.

A key strength of our study lies in its exploration of tissue-specific effects. Although the analysis of kidney cortex eQTLs did not reveal significant associations, autophagy-related genes associated with SLE risk were identified in skin tissues. Specifically, *TOLLIP*, *HSP90AB1*, and *BAG3* were implicated in non-sun-exposed skin, while *VAMP3*, *HSP90AB1*, *BAG1*, *BAG3*, and *STING1* were identified in sun-exposed skin. These findings are biologically plausible, as SLE is characterized by prominent skin involvement [27]. In addition, TOLLIP is known to participate in toll-like receptor (TLR) signaling pathways [28], while HSP90AB1 is involved in cellular stress responses and protein folding, processes that have been implicated in cutaneous immune regulation [29]. Together, these observations provide indirect support for the robustness and biological relevance of our findings. Notably, these skin-specific genetic signals were distinct from those discovered in blood and did not simply replicate the initial eQTL/pQTL findings. This distinction underscores the existence of tissue-specific mechanisms in SLE pathogenesis and highlights the critical importance of tissue context in genetic studies of complex diseases. However, these findings are based solely on computational analyses and lack clinical or experimental validation. Therefore, they should be interpreted with caution. Given that the skin is a frequently involved organ in SLE [30], these novel tissue-specific candidate genes may preliminary candidates for further functional investigation.

*NLRP6* encodes a protein involved in inflammasome formation [31]. Previous research has highlighted the role of *NLRP6* in regulating autophagy and inflammation. In inflammatory diseases including intracerebral hemorrhage [32] and inflammatory bowel disease [33], NLRP6 facilitates the clearance of damaged cellular components and excessive inflammasome activity to protect against tissue damage. Our study presents the inaugural genetic evidence establishing connections between *NLRP6* methylation and expression to SLE risk. We identified five methylation sites associated with disease susceptibility. Among these, four sites (cg06432119, cg08961832, cg09205751, and cg23437420) were associated with increased risk, while one site (cg12958961) was linked to reduced risk. Notably, four sites (cg06432119, cg08961832, cg09205751, and cg12958961) shared the same genetic variant with SLE, suggesting a potential role. Methylation has been shown to modulate inflammasome activity by regulating the expression and activation of inflammasome components, including *NLR* family members such as *NLRP6*. Hypermethylation of inflammasome genes suppresses their expression and activation [34]. This regulatory mechanism aligns with our findings, as higher methylation levels at cg06432119 were found in the PBMCs from SLE patients, along with reduced *NLRP6* expression. Future studies using more comprehensive approaches, such as MBD-seq or genome-wide bisulfite sequencing, are needed to better characterize the methylation landscape of *NLRP6* in SLE. Although *NLRP6* plays both pro- and anti-inflammatory roles [35], its anti-inflammatory function seems more relevant in SLE. Mechanistically, the downregulation of *NLRP6* may contribute to SLE risk by impairing the clearance of damaged cellular components and dysregulating inflammasome-mediated inflammatory responses, leading to sustained immune activation and tissue damage. This hypothesis finds support in existing evidence demonstrating that diminished NLRP6 function intensifies inflammatory responses through dysregulated inflammasome activity [36]. Thus, to bridge this gap and elucidate the precise mechanistic role of *NLRP6* in SLE pathogenesis, future studies employing both in vitro and in vivo models are warranted.

p27Kip1 is a CDK inhibitor that regulates cell cycle progression by inhibiting cyclin-CDK complexes, thereby maintaining cellular homeostasis and limiting uncontrolled cell proliferation. p27Kip1 regulates autophagy by being phosphorylated and stabilized through an AMPK-dependent process, which stimulates macroautophagy and contributes to cellular homeostasis [37]. AMPK activation in SLE has been shown to mitigate disease progression by inhibiting mTORC1 signaling, thereby reducing abnormal cell proliferation and autophagy dysregulation [38]. The interplay between AMPK and p27Kip1 in regulating both cell cycle and autophagy suggests that AMPK-driven stabilization of p27Kip1 could be a potential mechanism underlying the protective effects observed in SLE, as evidenced by the negative association between p27Kip1 and SLE risk. In addition, SNP rs34330 in *CDKN1B*, which has been linked to lower gene expression and disruptions in cell cycle regulation and immune tolerance [39], further aligns with our findings, suggesting that reduced *p27Kip1* levels may contribute to increased disease susceptibility. Moreover, reduced expression of *p27Kip1* was discovered in the PBMCs from SLE patients, confirming the negative association between its protein level and SLE risk as identified by SMR analysis. This further supports the protective role of *p27Kip1* in SLE.

In contrast with *NLRP6*, which had multiple methylation sites correlated with SLE risk but lacked external validation, both *HSPB8* and *WDR45L* showed significant methylation sites validated in external datasets, albeit without colocalization evidence. This highlights their potential associations with SLE, which may be driven by linkage disequilibrium rather than true variants, but further research is warranted to elucidate the underlying mechanisms and establish more definitive causal relationships. It is important to note that for *HSPB8* and *WDR45L*, sufficient or significant eQTL and pQTL data were not available or did not pass our initial SMR screening criteria in the discovery phase for blood-based analyses, precluding a comprehensive assessment of how their gene expression or protein abundance might influence SLE risk through SMR. Therefore, our understanding of their role is currently primarily informed by their methylation patterns. HSPB8 (heat shock protein family b member 8) is a small heat shock protein known for its role in protein quality control, particularly through chaperone-assisted selective autophagy. It interacts with BAG3 to target misfolded or damaged proteins for autophagic degradation, a process critical for maintaining cellular homeostasis [40]. In rheumatoid arthritis [41] and multiple sclerosis [42], *HSPB8* has been implicated in modulating inflammation, with evidence showing its involvement in dendritic cell maturation and pro-inflammatory cytokine release. This suggests that *HSPB8*’s role in autophagy extends beyond protein degradation, potentially influencing immune responses and contributing to the pathogenesis of autoimmune conditions like SLE. Nonetheless, the expression of *HSPB8* was comparable in the PBMC between control and SLE patients. On the other hand, *WDR45L* (WD repeat domain 45 like) encodes a protein that is part of the tryptophan–aspartic acid (WD) repeat protein family, which is involved in autophagy. Although *WDR45L* has not been extensively studied in SLE or other autoimmune diseases, its involvement in autophagy, particularly in regulating autophagosome biogenesis and maintaining endoplasmic reticulum homeostasis [43], suggests that it may influence immune homeostasis, which is often disrupted in SLE. The positive association between *WDR45L* methylation and SLE risk points to its potential role in disease pathogenesis, possibly through the regulation of autophagic pathways. Similarly, its expression was comparable between control group and SLE patients. This could be due to the cell type-specific expression profiles of *HSPB8* and *WDR45L* in peripheral blood.

This study has several limitations that should be considered. First, key blood-based eQTL (*NLRP6*) and pQTL (p27Kip1) findings were not replicated in independent datasets. This inconsistency may be attributed to differences in statistical power, genuine population heterogeneity, and variability in SLE phenotype definitions across GWAS resources. Nonetheless, the lack of statistical replication does not preclude biological relevance. Our pilot validation in clinical samples confirmed dysregulated NLRP6 expression in SLE cohorts, providing functional support that complements the genetic findings. Importantly, NLRP6 levels correlated inversely with SLEDAI even in the context of ongoing immunosuppressive therapy, implying that its expression may be more strongly governed by intrinsic disease state than by treatment-induced immunomodulation. These findings provide functional support complementing the genetic analyses, though they remain exploratory given the modest sample size. Second, limitations in data resources were notable. The discovery cohort’s sample size (1311 patients and 1783 controls) was relatively modest, although this dataset represents one of the most suitable publicly available resources for SMR analysis with well-matched QTL data. It is worth noting that effect sizes identified in smaller discovery datasets may be inflated, which may partially explain the reduced reproducibility in larger validation cohorts. In addition, the GTEx database has constraints in tissue-specific sample size and may not fully mirror in vivo disease states due to its post-mortem origin. Moreover, the absence of NLRP6 in the pQTL dataset further precluded a comprehensive analysis of its methylation-expression-protein axis. Therefore, given the limited coverage of current pQTL resources, the association between NLRP6 and SLE at the protein level remains speculative. Finally, while our integrative analysis identified skin-specific associations and repurposing candidates for HSPB8, these findings are preliminary. The tissue-specific signals require independent validation and functional studies, and the drug repurposing predictions, being based solely on transcriptional signatures, necessitate extensive experimental validation to ascertain their therapeutic potential. Future research should first seek to validate our novel findings—particularly the tissue-specific genetic associations in skin and the mQTLs for *HSPB8* and *WDR45L*—in larger, more diverse SLE cohorts. Moreover, functional studies are imperative to delineate the precise mechanistic roles of these genes, including *NLRP6*, in disease-relevant cell types.

## 5. Conclusions

In conclusion, by integrating large-scale multi-omics SMR analysis with preliminary clinical validation, our study prioritizes *NLRP6* as a candidate autophagy-related gene in SLE. Collectively, the observed hypermethylation, consequent downregulation of *NLRP6* expression, and its inverse correlation with disease activity support its potential pathogenic role and position it as a promising novel therapeutic avenue for SLE.

## Figures and Tables

**Table 1:** mQTL-eQTL integrative SMR analysis results for *NLRP6* methylation sites and gene expression.

Expo_probe	Outco_Gene	beta_SMR	se_SMR	*p*_SMR	Multi_*p*-Value_SMR	FDR	*p*_HEIDI
cg06432119	NLRP6	−0.778541	0.130664	2.5 × 10^−9^	2.5 × 10^−9^	3.89396 × 10^−8^	0.013
cg09205751	NLRP6	−0.735465	0.0987828	9.7 × 10^−14^	9.7 × 10^−14^	2.52088 × 10^−12^	0.024
cg23437420	NLRP6	−0.739102	0.107263	5.6 × 10^−12^	5.6 × 10^−12^	1.19638 × 10^−10^	0.077

## Data Availability

All data generated or analysed during this study are included in this published article.

## Supplementary Materials

The following supporting information can be downloaded at: https://www.mdpi.com/article/10.3390/genes17040466/s1, Figure S1: Manhattan plots and locus plots for QTL associations with SLE risk. (A) Manhattan plots for mQTL (upper) and eQTL (lower) associations with SLE risk. The orange dashed line indicates the significance threshold (*p*_SMR_multi = 0.05). *NLRP6* (cg06432119, cg09205751, cg23437420) methylation and expression were highlighted. (B, C) SMR locus plot demonstrates the significant association of *NLRP6* (cg06432119, cg09205751, cg23437420) methylation (B) and expression (C) with SLE risk. The x-axis represents the chromosomal positions of the genetic variants, and the y-axis represents the -log10 transformed *p*-values of the SMR test. The red dashed horizontal lines indicate the significance threshold (*p*_SMR_multi = 0.05). Figure S2: Effect plots of *NLRP6* associations with SLE. The red triangle represents the top cis-QTL, while the blue circles denote other cis-QTLs. The dashed orange line represents the linear regression fit for the effect sizes. Table S1. The full list for the 700 autophagy-related genes. Table S2. GWAS data sources and information for SLE discovery and replication cohorts. Table S3. Primers sequences for NLRP6 methylation loci. Table S4. Primers sequences for RT-qPCR analysis. Table S5. Complete SMR analysis results of blood mQTLs with SLE risk in the IEU discovery dataset. Table S6. Complete SMR analysis results of blood mQTLs with SLE risk in the FinnGen replication dataset. Table S7. Complete SMR analysis results of blood mQTLs with SLE risk in the GWAS Catalog replication dataset. Table S8. Validated mQTL analysis results for HSPB8 and WDR45L in SLE replication datasets. Table S9. Complete SMR analysis results of blood eQTLs with SLE risk in the IEU discovery dataset. Table S10. Complete SMR analysis results of blood eQTLs with SLE risk in the FinnGen replication dataset. Table S11. Complete SMR analysis results of blood eQTLs with SLE risk in the GWAS Catalog replication dataset. Table S12. Complete SMR analysis results of blood pQTLs with SLE risk in the IEU discovery dataset. Table S13. Complete SMR analysis results of blood pQTLs with SLE risk in the FinnGen replication dataset. Table S14. Complete SMR analysis results of blood pQTLs with SLE risk in the GWAS Catalog replication dataset. Table S15. Complete results of integrative SMR analyses between blood mQTLs and eQTLs. Table S16. Statistical Power of Mendelian Randomization Analyses for NLRP6 and CDKN1B. Table S17. Complete SMR analysis results of autophagy-related gene expression in Kidney Cortex tissue (GTEx v8) with SLE risk from the IEU discovery dataset. Table S18. Complete SMR analysis results of autophagy-related gene expression in Skin—Not Sun-Exposed (Suprapubic) tissue (GTEx v8) with SLE risk from the IEU discovery dataset. Table S19. Complete SMR analysis results of autophagy-related gene expression in Skin—Sun-Exposed (Lower leg) tissue (GTEx v8) with SLE risk from the IEU discovery dataset. Table S20. Predicted drug compounds targeting HSPB8 based on DSigDB analysis in Enrichr. Table S21. Personal information for samples included in this study.

## References

[1] Moulton V.R., Suarez-Fueyo A., Meidan E., Li H., Mizui M., Tsokos G.C. (2017). Pathogenesis of human systemic lupus erythematosus: A cellular perspective. Trends Mol. Med..

[2] Al-Gahtani S.N. (2021). A review of systemic lupus erythematosus (SLE): Symptoms, risk factors, treatment, and health related quality of life issues. Open J. Rheumatol. Autoimmune Dis..

[3] Li S., Gong T., Peng Y., Nieman K., Gilbertson D. (2020). Prevalence and incidence of systemic lupus erythematosus and associated outcomes in the 2009–2016 US Medicare population. Lupus.

[4] Barber M.R., Drenkard C., Falasinnu T., Hoi A., Mak A., Kow N.Y., Svenungsson E., Peterson J., Clarke A.E., Ramsey-Goldman R. (2021). Global epidemiology of systemic lupus erythematosus. Nat. Rev. Rheumatol..

[5] Zen M., Salmaso L., Amidei C.B., Fedeli U., Bellio S., Iaccarino L., Doria A., Saia M. (2023). Mortality and causes of death in systemic lupus erythematosus over the last decade: Data from a large population-based study. Eur. J. Intern. Med..

[6] Hoi A.Y., Morand E.F. (2021). Treatment update in systemic lupus erythematous. Rheum. Dis. Clin..

[7] Nimesh S., Ahmad M.I., Dhama S., Kumar P., Akram M., Hasaroeih N.E.N. (2021). Systemic Lupus Erythematosus Disease: An Overview of the Clinical Approach to Pathogenesis, Diagnosis, and Treatment. Borneo J. Pharm..

[8] Yang Z., Goronzy J.J., Weyand C.M. (2015). Autophagy in autoimmune disease. J. Mol. Med..

[9] Ciccacci C., Perricone C., Alessandri C., Latini A., Politi C., Delunardo F., Pierdominici M., Conti F., Novelli G., Ortona E. (2018). Evaluation of ATG5 polymorphisms in Italian patients with systemic lupus erythematosus: Contribution to disease susceptibility and clinical phenotypes. Lupus.

[10] Caza T., Wijewardena C., Al-Rabadi L., Perl A. (2022). Cell type-specific mechanistic target of rapamycin-dependent distortion of autophagy pathways in lupus nephritis. Transl. Res..

[11] Colasanti T., Spinelli F.R., Barbati C., Ceccarelli F., Scarpa S., Vomero M., Alessandri C., Valesini G., Conti F. (2022). Belimumab decreases autophagy and citrullination in peripheral blood mononuclear cells from patients with systemic lupus erythematosus. Cells.

[12] Nikoleri D., Semitekolou M., Gkirtzimanaki A., Georgakis S., Verginis P., Sidiropoulos P., Bertsias G. (2024). P17 Autophagy-based unconventional secretion of B cell activating factor (BAFF) by monocytes in Systemic Lupus Erythematosus (SLE). Lupus Sci. Med..

[13] Podestà M.A., Faravelli I., Ponticelli C. (2022). Autophagy in lupus nephritis: A delicate balance between regulation and disease. Autoimmun. Rev..

[14] Yang H., Liu D., Zhao C., Feng B., Lu W., Yang X., Xu M., Zhou W., Jing H., Yang J. (2021). Mendelian randomization integrating GWAS and eQTL data revealed genes pleiotropically associated with major depressive disorder. Transl. Psychiatry.

[15] Liu X., Miao Y., Liu C., Lu W., Feng Q., Zhang Q. (2023). Identification of multiple novel susceptibility genes associated with autoimmune thyroid disease. Front. Immunol..

[16] Wu Y., Zeng J., Zhang F., Zhu Z., Qi T., Zheng Z., Lloyd-Jones L.R., Marioni R.E., Martin N.G., Montgomery G.W. (2018). Integrative analysis of omics summary data reveals putative mechanisms underlying complex traits. Nat. Commun..

[17] Võsa U., Claringbould A., Westra H.-J., Bonder M.J., Deelen P., Zeng B., Kirsten H., Saha A., Kreuzhuber R., Yazar S. (2021). Large-scale cis-and trans-eQTL analyses identify thousands of genetic loci and polygenic scores that regulate blood gene expression. Nat. Genet..

[18] Ferkingstad E., Sulem P., Atlason B.A., Sveinbjornsson G., Magnusson M.I., Styrmisdottir E.L., Gunnarsdottir K., Helgason A., Oddsson A., Halldorsson B.V. (2021). Large-scale integration of the plasma proteome with genetics and disease. Nat. Genet..

[19] Giambartolomei C., Vukcevic D., Schadt E.E., Franke L., Hingorani A.D., Wallace C., Plagnol V. (2014). Bayesian test for colocalisation between pairs of genetic association studies using summary statistics. PLoS Genet.

[20] Rasooly D., Peloso G.M., Giambartolomei C. (2022). Bayesian Genetic Colocalization Test of Two Traits Using coloc. Curr. Protoc..

[21] Pairo-Castineira E., Rawlik K., Bretherick A.D., Qi T., Wu Y., Nassiri I., McConkey G.A., Zechner M., Klaric L., Griffiths F. (2023). GWAS and meta-analysis identifies 49 genetic variants underlying critical COVID-19. Nature.

[22] Chen E.Y., Tan C.M., Kou Y., Duan Q., Wang Z., Meirelles G.V., Clark N.R., Ma’ayan A. (2013). Enrichr: Interactive and collaborative HTML5 gene list enrichment analysis tool. BMC Bioinform..

[23] Kuleshov M.V., Jones M.R., Rouillard A.D., Fernandez N.F., Duan Q., Wang Z., Koplev S., Jenkins S.L., Jagodnik K.M., Lachmann A. (2016). Enrichr: A comprehensive gene set enrichment analysis web server 2016 update. Nucleic Acids Res.

[24] Xie Z., Bailey A., Kuleshov M.V., Clarke D.J.B., Evangelista J.E., Jenkins S.L., Lachmann A., Wojciechowicz M.L., Kropiwnicki E., Jagodnik K.M. (2021). Gene Set Knowledge Discovery with Enrichr. Curr. Protoc..

[25] Yoo M., Shin J., Kim J., Ryall K.A., Lee K., Lee S., Jeon M., Kang J., Tan A.C. (2015). DSigDB: Drug signatures database for gene set analysis. Bioinformatics.

[26] Zhu Z., Zhang F., Hu H., Bakshi A., Robinson M.R., Powell J.E., Montgomery G.W., Goddard M.E., Wray N.R., Visscher P.M. (2016). Integration of summary data from GWAS and eQTL studies predicts complex trait gene targets. Nat. Genet..

[27] Stull C., Sprow G., Werth V.P. (2023). Cutaneous Involvement in Systemic Lupus Erythematosus: A Review for the Rheumatologist. J. Rheumatol..

[28] Sun L., Liu W., Zhang L.J. (2019). The Role of Toll-Like Receptors in Skin Host Defense, Psoriasis, and Atopic Dermatitis. J. Immunol. Res..

[29] Prodromou C. (2016). Mechanisms of Hsp90 regulation. Biochem. J..

[30] Nakabo S., Romo-Tena J., Kaplan M.J. (2022). Neutrophils as Drivers of Immune Dysregulation in Autoimmune Diseases with Skin Manifestations. J. Invest. Dermatol..

[31] Zheng D., Kern L., Elinav E. (2021). The NLRP6 inflammasome. Immunology.

[32] Xiao H., Chen H., Jiang R., Zhang L., Wang L., Gan H., Jiang N., Zhao J., Zhai X., Liang P. (2020). NLRP6 contributes to inflammation and brain injury following intracerebral haemorrhage by activating autophagy. J. Mol. Med..

[33] Fan W., Ding C., Liu S., Gao X., Shen X., De Boevre M., Gao Z., Li M., Zhang S., Miao Y. (2022). Estrogen receptor β activation inhibits colitis by promoting NLRP6-mediated autophagy. Cell Rep..

[34] Yi Y.-S. (2021). Functional interplay between methyltransferases and inflammasomes in inflammatory responses and diseases. Int. J. Mol. Sci..

[35] Ghimire L., Paudel S., Jin L., Jeyaseelan S. (2020). The NLRP6 inflammasome in health and disease. Mucosal Immunol..

[36] Wlodarska M., Thaiss C.A., Nowarski R., Henao-Mejia J., Zhang J.P., Brown E.M., Frankel G., Levy M., Katz M.N., Philbrick W.M. (2014). NLRP6 inflammasome orchestrates the colonic host-microbial interface by regulating goblet cell mucus secretion. Cell.

[37] Qi Y.y., Zhou X.j., Zhang H. (2019). Autophagy and immunological aberrations in systemic lupus erythematosus. Eur. J. Immunol..

[38] He J., Ma J., Ren B., Liu A. (2020). Advances in systemic lupus erythematosus pathogenesis via mTOR signaling pathway. Semin. Arthritis Rheum..

[39] Yang W., Tang H., Zhang Y., Tang X., Zhang J., Sun L., Yang J., Cui Y., Zhang L., Hirankarn N. (2013). Meta-analysis followed by replication identifies loci in or near CDKN1B, TET3, CD80, DRAM1, and ARID5B as associated with systemic lupus erythematosus in Asians. Am. J. Hum. Genet..

[40] Cristofani R., Piccolella M., Crippa V., Tedesco B., Montagnani Marelli M., Poletti A., Moretti R.M. (2021). The role of HSPB8, a component of the chaperone-assisted selective autophagy machinery, in cancer. Cells.

[41] Roelofs M.F., Boelens W.C., Joosten L.A., Abdollahi-Roodsaz S., Geurts J., Wunderink L.U., Schreurs B.W., van den Berg W.B., Radstake T.R. (2006). Identification of small heat shock protein B8 (HSP22) as a novel TLR4 ligand and potential involvement in the pathogenesis of rheumatoid arthritis. J. Immunol..

[42] Peferoen L.A., Gerritsen W.H., Breur M., Ummenthum K.M., Peferoen-Baert R.M., van der Valk P., van Noort J.M., Amor S. (2015). Small heat shock proteins are induced during multiple sclerosis lesion development in white but not grey matter. Acta Neuropathol. Commun..

[43] Tornero-Écija A., Tábara L.-C., Bueno-Arribas M., Antón-Esteban L., Navarro-Gómez C., Sánchez I., Vincent O., Escalante R. (2022). A Dictyostelium model for BPAN disease reveals a functional relationship between the WDR45/WIPI4 homolog Wdr45L and Vmp1 in the regulation of autophagy-associated PtdIns3P and ER stress. Autophagy.

