# Supplementary figures and images for: Multi-Omics Mendelian Randomization and Clinical Validation Implicate NLRP6 as a Candidate Autophagy-Related Gene in Systemic Lupus Erythematosus
Source: Genes (Basel). 2026 Apr 16;17(4):466. doi: 10.3390/genes17040466 (PMC13115566; doi:10.3390/genes17040466)

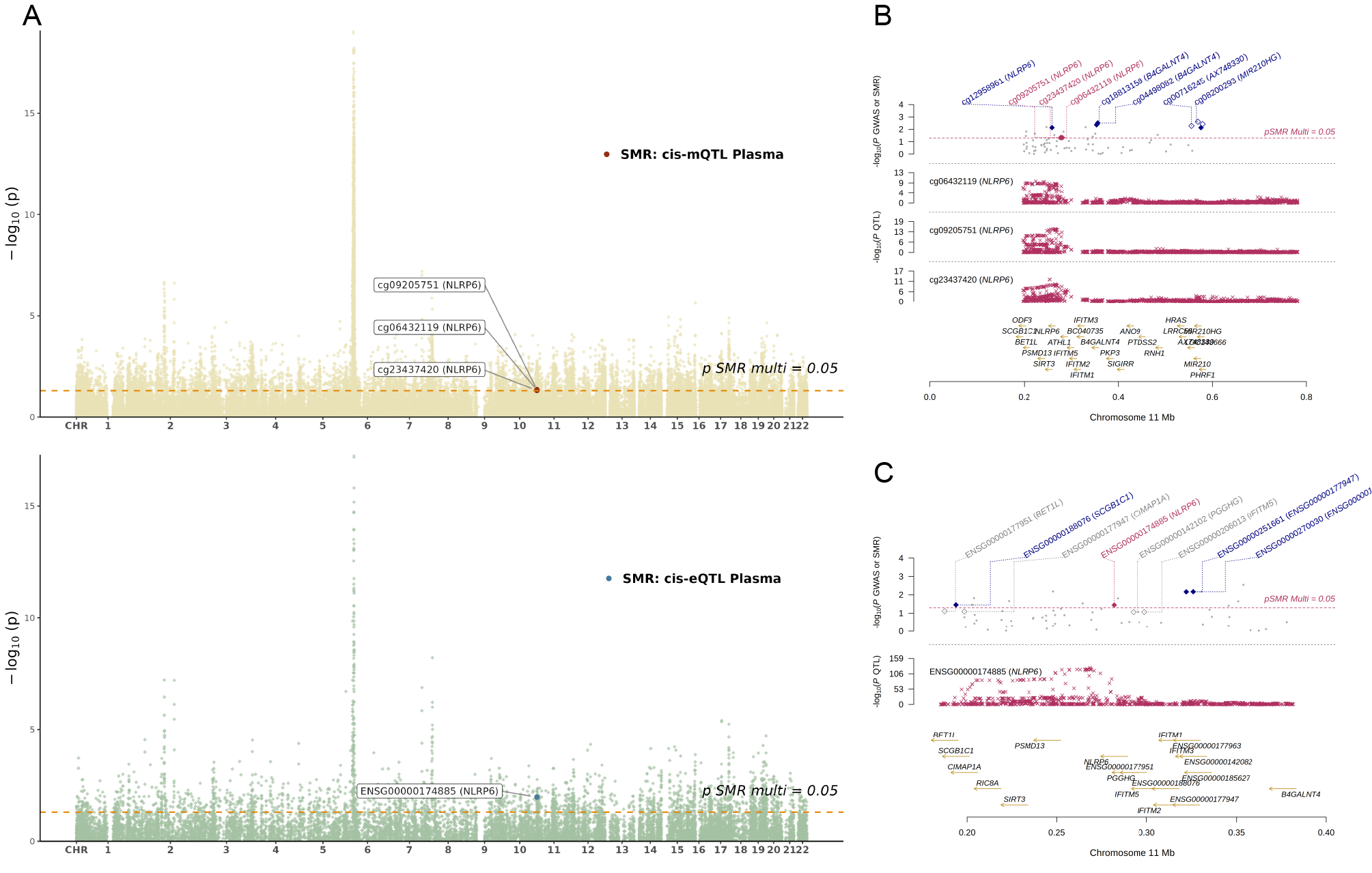

Supplement: Supplementary file 1 [file genes-17-00466-s001.zip › Figure S1.tif]

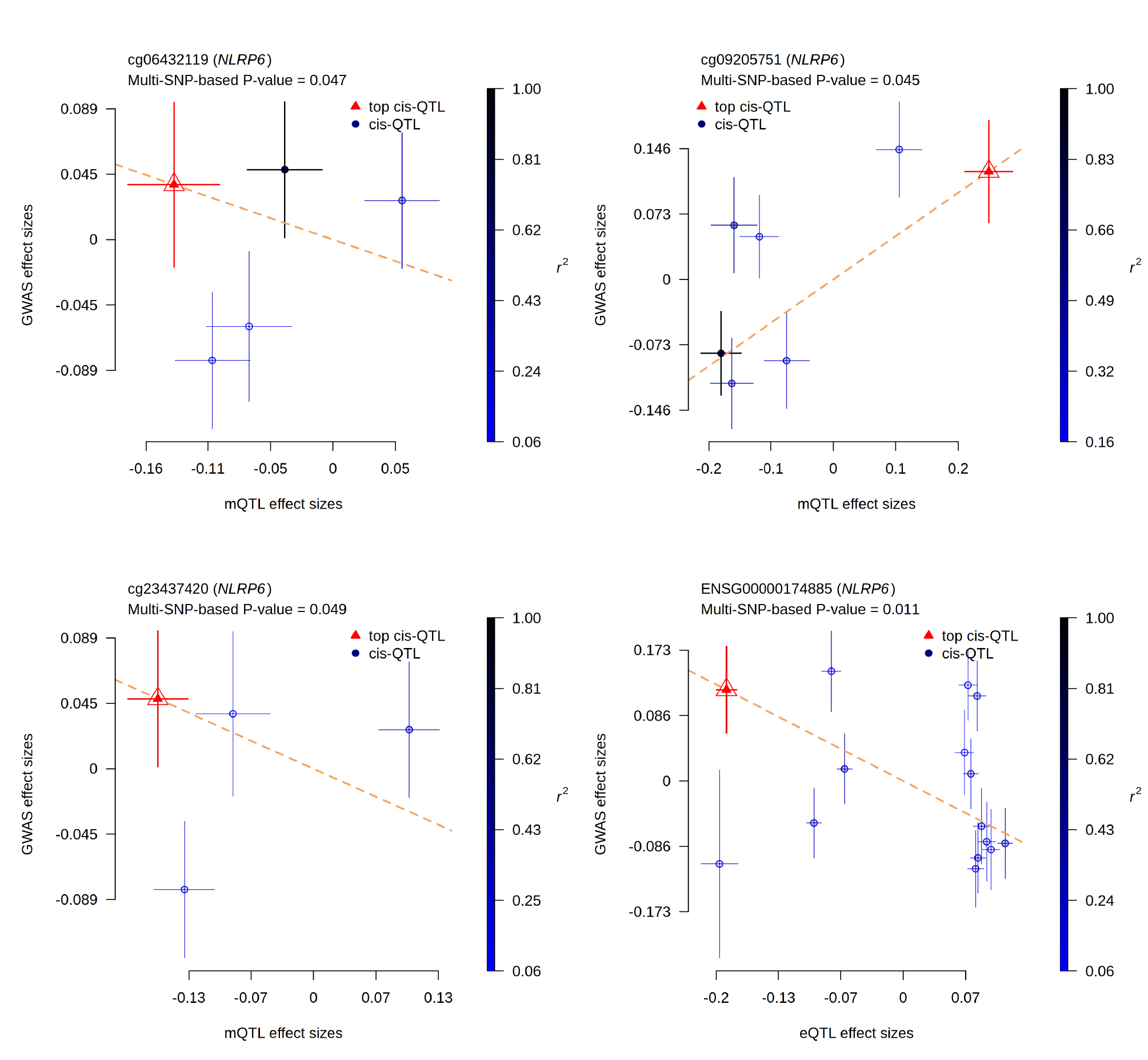

Supplement: Supplementary file 1 [file genes-17-00466-s001.zip › Figure S2.tif]
